# Antiarthritic and Antihyperalgesic Properties of Ethanolic Extract from *Gomphrena celosioides* Mart. (Amaranthaceae) Aerial Parts

**DOI:** 10.1155/2020/4170589

**Published:** 2020-09-15

**Authors:** Luis Fernando Benitez Macorini, Joyce Alencar Santos Radai, Rafael Souza Maris, Saulo Euclides Silva-Filho, Maicon Matos Leitao, Sérgio Faloni de Andrade, Dayanna Isabel Araque Gelves, Marcos Jose Salvador, Arielle Cristina Arena, Cândida Aparecida Leite Kassuya

**Affiliations:** ^1^School of Health Sciences, Federal University of Grande Dourados (UFGD), Dourados, MS, Brazil; ^2^School of Health Sciences, University Center of Grande Dourados (UNIGRAN), Dourados, MS, Brazil; ^3^College of Pharmaceutical Sciences, Food and Nutrition, Federal University of Mato Grosso do Sul (UFMS), Campo Grande, MS, Brazil; ^4^Research Center for Biosciences and Health Technologies (CBIOS), Lusofona University, Lisbon, Portugal; ^5^Institute of Biology, Department of Plant Biology, PPG BTPB, PPG BCE, University of Campinas (UNICAMP), Campinas, São Paulo, Brazil Institute of Biology, Brazil; ^6^Department of Structural and Functional Biology, Institute of Biosciences of Botucatu, UNESP—University Estadual Paulista—Botucatu, São Paulo State, Botucatu, Brazil

## Abstract

*Gomphrena celosioides* Mart. (Amaranthaceae) is used in folk medicine as a natural analgesic, and in Brazil, the species of genus *Gomphrena* is used for rheumatism. However, scientific evidence which supports its popular use as an analgesic is scarce. This study assessed the antiarthritic and antihyperalgesic activities of the ethanolic extract obtained from *G*. *celosioides* aerial parts on Swiss or *C57BL/6* mice. The antiarthritic and antihyperalgesic potential of *Gomphrena celosioides* was evaluated using paw edema, mechanical hyperalgesia, cold allodynia, carrageenan-induced pleurisy, articular inflammation zymosan-induced, Freund's complete adjuvant-induced inflammation zymosan-induced peritonitis, and carrageenan-induced adhesion and rolling experiment models. All doses of *G*. *celosioides* (300, 700, and 1000 mg/kg) significantly reduced edema formation in all the intervals evaluated, whereas the mechanical hyperalgesia was reduced 3 hours after the carrageenan injection. The cold hyperalgesia was significantly decreased 3 (700 mg/kg) and 4 hours (700 and 1000 mg/kg) after the carrageenan injection. Ethanolic extract of *G*. *celosioides* at 1000 mg/kg reduced the total leukocyte number, without interfering in the protein extravasation in carrageenan-induced pleurisy model. Ethanolic extract of *G*. *celosioides* (300 mg/kg) was also able to reduce significantly the leukocyte migration in zymosan-induced articular edema, while a reduction of the adhesion and migration and leukocyte rolling was induced by the ethanolic extract of *G*. *celosioides* (300 mg/kg) in zymosan-induced peritonitis. In Freund's complete adjuvant-induced inflammation model, an edema formation and mechanical hyperalgesia reduction were induced by the ethanolic extract of *G*. *celosioides* on day 22, whereas the cold allodynia was reduced on day 6 of treatment with the extract. These results show that ethanolic extract of *G*. *celosioides* has antihyperalgesic and antiarthritic potential in different acute and persistent models, explaining, at least in part, the ethnopharmacological relevance of this plant as a natural analgesic agent.

## 1. Introduction

Scientific evidence has demonstrated that products from natural sources, including medicinal plants, are promising for the development of safe alternatives for the treatment of pain management and inflammatory diseases [1]. Thus, the ethnopharmacologically guided research has contributed with the identification of new therapeutic agents obtained from plants [2], which often have fewer adverse effects, and is important for patients who use medications for long periods.

*Gomphrena celosioides* Mart. (synonyms *G*. *serrata and G*. *decumbens*), an annual herbknown popularly as “Perpétua Brava,” belongs to the Amaranthaceae family [3] and can be found in America, Australia, and Indomalaysia. In Brazil, this species occurs in savanna vegetation, napeadic grassland, high altitude grassland, and caatinga [4]. This plant is used for several folk medicinal purposes, such as for the treatment of several liver-related and dermatological diseases, dysmenorrhea, bronchial infections, renal disorders, and also as an analgesic [4–8].

Several chemical compounds with high therapeutic potential, such as hydrocarbons, alcohol, steroids, terpenes, ecdysteroids, flavonoids, saponins, butacyanine, and ketoses, have already been isolated from *G*. *celosioides* [9]. De Moura et al. [10] identified and isolated chemical compounds from *G*. *celosioides* aerial parts, including vanillic acid, 4-hydroxy-benzoic acid, and 4-hydroxy-3-methoxybenzoic acid, in addition to stigmasterol, sitosterol, and campesterol. Dosumu et al. [11] identified and isolated 3-(4-hydroxyphenyl)methylpropenoate from the methanol extract of *G*. *celosioides*. These same authors also found aurantiamide and aurantiamide acetate from the *n*-hexane extract of *G*. *celosioides* [12].

Despite its importance in folk medicine, there are few scientific studies which validate its therapeutic effects, especially the analgesic activity. Some studies using the aerial parts of *G*. *celosioides* extract have already reported its antihypertensive [8], antitumor, antimicrobial [10], cytotoxic, anti-inflammatory, and analgesic properties [13]. In a study carried out by Vasconcelos et al. [8], the ethanolic extract of *G*. *celosioides* showed diuretic effect and reduced the blood pressure in rats, demonstrating potential as an antihypertensive drug. Oluwabunmi and Abiola [6] showed a gastroprotective effect of the methanolic extract obtained from leaves, while De Moura et al. [10] found an antimicrobial effect of the crude extract of the plant against *Staphylococcus aureus* and *Salmonella typhi*. In another study, the ethyl acetate and methanol extracts were active against *Fasciola gigantica, Taenia solium*, and *Pheretima posthuma,* corroborating the popular use of *G*. *celosioides* in the treatment of infectious diseases [11].

Although *G*. *celosioides* is a species widely used in folk medicine with important bioactive compounds, few scientific studies are found in the literature to confirm its popular indication, especially regarding its antiarthritic and antihyperalgesic potential. Thus, this study aimed to evaluate the analgesic and antiarthritic activities of the ethanolic extract of the *G*. *celosioides* aerial parts in different acute and persistent inflammation models.

## 2. Materials and Methods

### 2.1. Plant Material and Preparation of Ethanolic Extract

*G*. *celosioides* aerial parts were collected (lat: −19.666667; long: −51.183333 WGS84) and identified by Dr. Josafá Carlos de Siqueira. A voucher specimen (SCAB 4051) is deposited in the herbarium of Pontifical Catholic University, Rio de Janeiro. The preparation of ethanolic extract of *G*. *celosioides* (EEGC) was performed according to Vasconcelos et al. [8].

### 2.2. Animals

**Figure 1:**
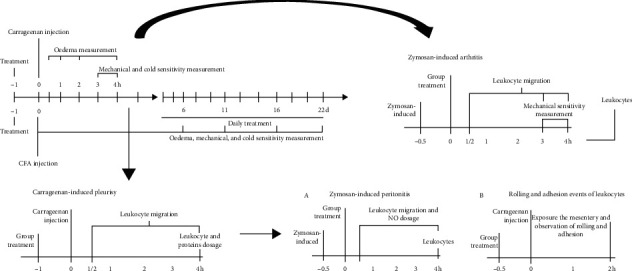
The experimental design.

Male and female *Swiss* or *C57BL6* mice (weighing 20–30 g; 60–65 days of age) were provided by the Central Animal House of the Federal University of Grande Dourados/Mato Grosso do Sul, Brazil. The animals were housed at 22 ± 2°C under a 12/12 h light/dark cycle with free access to food and water. Prior to the experiments, the animals were fasted overnight, with water provided *ad libitum*. The experimental protocols were in accordance with the Ethical Principles in Animal Research adopted by the Brazilian College of Animal Experimentation and were approved by the Ethical Committee in Animal Experimentation of the Federal University of Grande Dourados (protocol number: 09/2018). The experimental design is shown in Figure 1.

### 2.3. Reagents

Carrageenan, dexamethasone, zymosan, indomethacin, acetone, and Bradford reagent were purchased from Sigma-Aldrich Co. LLC. (St. Louis, MO, USA).

### 2.4. Paw Edema, Mechanical Hyperalgesia, and Cold Allodynia Induced by Carrageenan

*Swiss* male mice were allocated into five groups: negative control group (treated with saline 0.9%, p.o.), positive control group (Dexa; dexamethasone 1 mg/kg, s.c.), and three groups treated with different doses of ethanolic extract of *G*. *celosioides* (EEGC) (300, 700, or 1000 mg/kg, p.o.). One hour after the treatment, all animals received 50 *μ*L of carrageenan (300 *μ*g/paw, s.c.) in the right hind paw and saline solution in the left hind paw (used as a control). The paw volume was measured in time intervals (1, 2, and 4 h) using a plethysmometer device. Mechanical hyperalgesia was evaluated by the electronic Von Frey pressure-increasing test at time intervals 3 and 4 h [14]. Sensitivity to cold was performed by the acetone drop test described by Decosterd et al. [15], at time intervals 3 and 4 h. Acetone (30 *μ*L) was released over the right paw of the animals. Right after, the number of times in which the paw rising reaction occurred was evaluated. Minimum and maximum cutoff points were assigned at 5 and 20 s, respectively.

### 2.5. Model of Carrageenan-Induced Pleurisy in Mice

*Swiss* female mice (50 days of age) were treated and allocated into five groups: negative control group (saline solution 0.9% p.o.), positive control group (Dexa, 1 mg/kg, s.c.), and three groups treated with different doses of EEGC (300, 700, or 1000 mg/kg p.o.). One hour after the treatment, 1 mL of carrageenan (300 *μ*g/cavity, diluted in sterile saline) was injected into the animals by the intrapleural pathway, as described by Vinegar et al. [16]. After 4 h of the carrageenan injection, the animals were anesthetized and euthanized (ketamine/xylazine solution 1 : 1). The exudate was collected by aspiration and put into tubes. The leukocyte count was performed in the Neubauer chamber, and the total protein was determined by the Bradford method using a commercial kit Bioagency®.

### 2.6. Leukocyte Recruitment and Mechanical Hyperalgesia Evaluation in Experimental Model of Zymosan-Induced Arthritis

The experimental model of zymosan-induced arthritis was carried out as previously reported [17]. The right knee joints of the animals received 200 *μ*g/cavity of zymosan (in 10 *μ*L sterile saline; intra-articularly injected), while the contralateral knee joint received an equal volume of saline. Thirty minutes before zymosan injection, the mice were treated orally with vehicle (saline) or EEGC (300 mg/kg). The additional mice group received only saline in the articular cavity and was treated with vehicle (naive group). At times of 3 and 4 h after zymosan injection, the mechanical hyperalgesia was evaluated using a digital analgesimeter (Von Frey, Insight®), a pressure transducer which records the applied force (in grams) in paw until the moment of paw withdrawal. At a time of 6 h after zymosan injection, the animals were anesthetized and euthanatized, and the knee joint was exposed by surgical incision and washed twice with 5 *μ*L of phosphate-buffered saline (PBS) containing ethylenediaminetetraacetic acid (EDTA). The supernatant was diluted to a final volume of 50 *μ*L with PBS/EDTA to determine the total cells counts.

### 2.7. Zymosan-Induced Peritonitis

*Swiss* male mice were allocated into four groups: naive group (saline 0.9%, p.o.), negative control group (saline 0.9%, p.o.), positive control group (Dexa, 1 mg/kg s.c.), and EEGC (300 mg/kg, p.o.). Peritonitis was induced by 1 mg/kg of zymosan administrated intraperitoneally 30 min after the treatment in each animal [18]. The naive group received saline solution for control. The zymosan-induced peritonitis was assessed 6 h after the administration. After this period, the animals were euthanized, and their peritoneal cavity was washed with 1 mL of PBS/EDTA. Then, the solution containing the wash was centrifuged, and the supernatant was used for the nitric oxide (NO) dosage, and the precipitate was resuspended in 1 mL of PBS/EDTA for the total leukocyte analysis using the KX-21n Roche® equipment. The nitric oxide determination nitrite was measured by methods of Griess. A Griess solution was prepared, where 50 *μ*L of the solution and 50 *μ*L of the sample were added in a 96-well microplate; after a 15 min wait, the reading was performed in a spectrophotometer at 580 nm. A nitrite curve using sodium nitrite at 5, 10, 30, and 60 *μ*M concentrations was also performed [19].

### 2.8. In Situ Intravital Microscopy Analysis for Rolling and Adhesion Events of Leukocytes in the Mesenteric Microcirculation

The leukocyte rolling and adhesion were performed after the induction of leukocyte migration by an injection of carrageenan (500 *μ*g/cavity, i.p.) in sterile saline. The mice were treated orally with EEGC (300 mg/kg), vehicle (saline), or indomethacin (5 mg/kg) as a reference drug, 30 min before the carrageenan injection. The additional mice group was injected only with saline in the peritoneal cavity. After 2 h of carrageenan or saline injection, the animals were anesthetized (ketamine/xylazine solution 1 : 1). A lateral surgical incision was performed in the abdominal wall to the exposure of the mesentery and observation of *in situ* microcirculation. The mice were kept on a heated plate, with temperature maintained at 37°C, adapted to the chariot of an optical microscope with a video camera and monitor to project and record the images. The preparation was kept moist and warm with Ringer Locke's solution that contained 1% gelatin. The vessels considered the postcapillary venules with 10–18 *μ*m diameter. The number of rolling and adherent leukocytes was counted as 10 min intervals. Leukocyte adherence was determined when cells remained static in the endothelium for 30 s or more [20].

### 2.9. Paw Edema and Mechanical Hyperalgesia Induced by Freund's Complete Adjuvant (CFA)

The persistent model of edema and mechanical hyperalgesia induced by Freund's complete adjuvant (CFA) in male *C57BL6* mice was performed to study the analgesic and anti-inflammatory properties with prolonged treatment with EEGC. The animals were allocated into three groups: control group (saline 0.9%, p.o.), EEGC group (100 mg/kg, p.o.), and positive control group (Dexa, 1 mg/kg s.c.).

At time zero, 20 *μ*L of a suspension containing dead *Mycobacterium tuberculosis* and added in paraffin oil (85%) and monooleate (15%) was injected into the right hind paw. The nociceptive threshold was estimated at 3 and 4 h after CFA and was then analyzed on days 6, 11, 16, and 22 using the Von Frey electronic test [21]. In addition, CFA-induced edema was resolved at 2 and 4 h intervals on days 6, 11, 16, and 22 after CFA injection with a plethysmometer.

Cold sensitivity was measured by the acetone drop test as described by Eliav et al. [22]. A blind needle attached to a syringe was used to release 30 *μ*L of acetone in the paw of CFA animals from the CFA model experiment on days 6, 11, 16, and 22, and the duration (in *s*) of paw withdrawal was evaluated. The minimum and maximum cutoff points were assigned to be 0.5 and 20 s, respectively. Paw removals due to locomotion or weight change were not counted.

### 2.10. Statistical Analysis

The data are presented as the mean ± SEM (standard error of the mean). Differences among means were evaluated by one-way analysis of variance (ANOVA), followed by the Newman–Keuls post hoc test, using GraphPad Prism software. Statistical differences were considered significant when *P* < 0.05.

## 3. Results

### 3.1. Effects of EEGC on the Paw Edema, Mechanical Hyperalgesia, and Cold Allodynia Induced by Carrageenan

**Figure 2:**
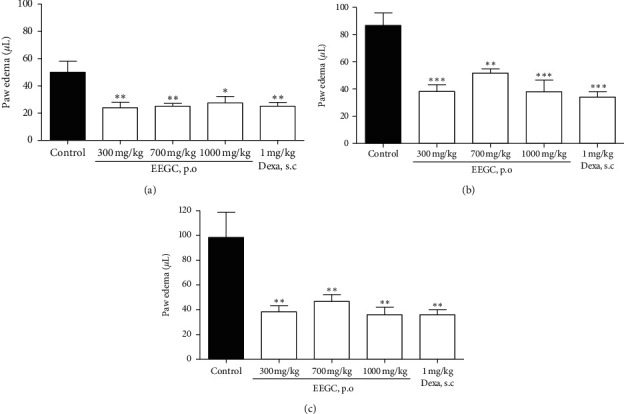
Effect of oral administration of EEGC at 1 (a), 2 (b), and 4 (c) hours after carrageenan-induced edema. The control (saline 0.9%, p.o.), EEGC (300, 700, or 1000 mg/kg, p.o.), and DEXA (dexamethasone 1 mg/kg, i.p.) groups were treated after 1 hour with carrageenan. The bars express the mean ± SEM compared to the control group. ^*∗*^*P* < 0.05; ^*∗∗*^*P* < 0.01; ^*∗∗∗*^*P* < 0.001. One-way analysis of variance followed by the Newman–Keuls test.

All doses of EEGC (300, 700, and 1000 mg/kg) reduced the edema formation in the first, second, and fourth hours after the carrageenan administration with a maximum inhibition of 61 ± 5%, 53 ± 6%, and 68 ± 5% for at 300, 700, and 1000 mg/kg, respectively. The values were similar to those of the animals treated with dexamethasone, which had its maximum antiedematogenic activity in the fourth hour reducing 68 ± 4% paw edema (Figures 2(a)–2(c)).

**Figure 3:**
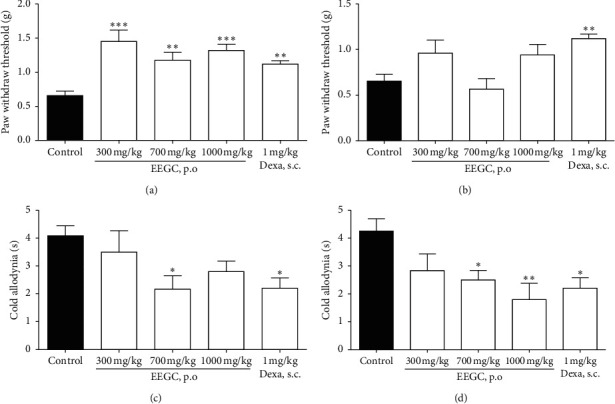
Effect of oral administration of EEGC at 3 h and 4 h after carrageenan-induced mechanical sensitivity (a, b) and cold hypersensitivity (c, d). The control (saline 0.9%, p.o.), EEGC (300, 700, or 1000 mg/kg, p.o.), and dexa (dexamethasone 1 mg/kg, s.c.) groups were treated after 1 hour with carrageenan. The bars express the mean ± SEM compared to the control group. ^*∗∗*^*P* < 0.01, ^*∗*^*P* < 0.05, and ^*∗∗∗*^*P* < 0.001. One-way analysis of variance followed by the Newman–Keuls test.

Furthermore, the treatment with all doses of extract (300, 700, and 1000 mg/kg) after 3 h of the carrageenan injection reduced the mechanical hyperalgesia (Figure 3). EEGC exhibited maximal activity on the mechanical hyperalgesia at 300 mg/kg with 91 ± 22%, a reduction similar to those observed with dexamethasone treatment (87 ± 8%). However, it was not possible to observe the same reduction after the fourth hour (Figures 3(a) and 3(b)). In relation to cold allodynia, EEGC treatment promoted a reduction at 700 mg/kg of EEGC in the third hour and in the fourth hour occurred the allodynia reduction in the doses of 700 and 1000 mg/kg with a maximum inhibition of 58 ± 14% (Figures 3(c) and 3(d)).

### 3.2. Effects of EEGC on Carrageenan-Induced Pleurisy

**Figure 4:**
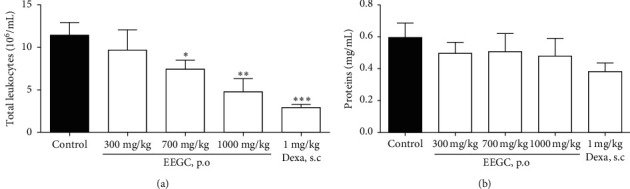
Effect of the oral administration of EEGC on acute inflammation induced by intrapleural injection of carrageenan in mice. In (a), leukocyte migration ×10^6^ cells/cavity and (b) proteins (mg/ml). The control group received saline solution (0.9%), and the EEGC groups received 300, 700, or 1000 mg/kg. The bars express the mean ± SEM compared to the control versus treated group. ^*∗*^*P* < 0.05; ^*∗∗*^*P* < 0.01, and ^*∗∗∗*^*P* < 0.001. One-way analysis of variance followed by the Newman–Keuls test.

The EEGC treatment at the dose of 300 mg/kg showed a significant reduction (58 ± 14%) of the leukocyte migration compared to the control group, indicating a possible reduction of the inflammatory process (Figure 4(a)). However, the treatment with the extract did not show reduction in the protein extravasation to the pleural cavity (Figure 4(b)).

### 3.3. Effects of EEGC on Zymosan-Induced Articular Inflammation and Peritonitis

**Figure 5:**
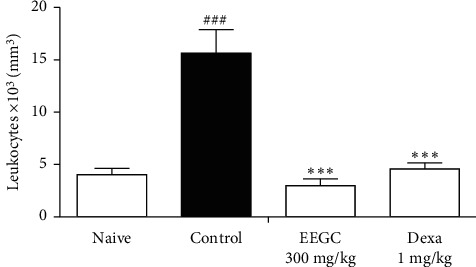
Effect of the oral administration of EEGC on the leukocyte recruitment in articular inflammation induced by the zymosan model in mice. The figures show the values after the induction of arthritis in the naive, control (saline, 0.9%, p.o.), EEGC (300 mg/kg, p.o.), and dexa (dexamethasone 1 mg/kg, s.c.) groups. The bars express the mean ± SEM. ^###^ or ^*∗∗∗*^*P* < 0.001. ^###^Control versus naïve. ^*∗∗∗*^EEGC versus control. One-way analysis of variance followed by the Newman–Keuls test.

**Figure 6:**
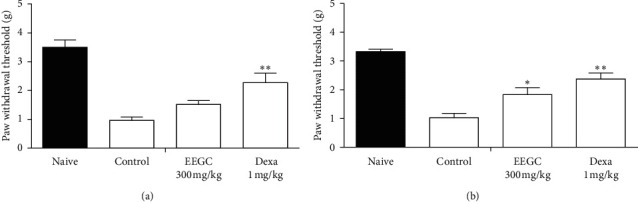
Effect of the oral administration of EEGC on the increase in mechanical sensitivity (paw withdrawal threshold) in articular inflammation induced by the zymosan model in mice. The figures show the values at 3 (a) and 4 h (b) after the procedure of induction of articular inflammation in the naive, control up (saline 0.9%, p.o.), EEGC (100 mg/kg, p.o.), and dexa (1 mg/kg, s.c.) groups. The bars express the mean ± SEM compared with the control vs. treated group. ^*∗*^*P* < 0.05; ^*∗∗*^*P* < 0.01; One-way analysis of variance followed by the Newman–Keuls test.

**Figure 7:**
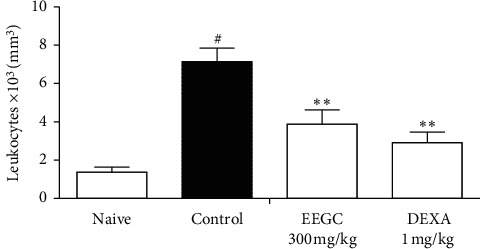
Effect of the oral administration of EEGC on leukocyte migration after peritoneal injection of carrageenan in mice. Mice were pretreated with EEGC (300 mg/kg, p.o.), dexamethasone (dexa, 1 mg/kg, i.p.), or vehicle (saline solution, 0.9%, p.o.). After 60 min, naive mice were injected with saline i.p., while all other groups received zymosan. The bars express the mean ± SEM compared with the control vs. treated group; ^#^ or ^*∗∗*^*P* < 0.001. ^#^Control versus naive. ^*∗∗*^EEGC versus control. One-way analysis of variance followed by the Newman–Keuls test.

In another model of articular inflammation, EEGC treatment at a dose of 300 mg/kg promoted a reduction of hyperalgesia and leukocyte migration compared to the control group with a maximum inhibition of 52 ± 3% and 81 ± 4%, respectively (Figures 5 and 6). There was a significant reduction of 46 ± 10% induced by EEGC in the total leukocytes migration in peritonitis at 300 mg/kg dose (Figure 7); therefore, EEGC did not alter significantly the nitric oxide (NO) levels (figure not shown). The dexamethasone group inhibited significantly the hyperalgesia and leukocyte migration in articular injection (Figures 5 and 6) and also the leukocyte migration in peritonitis (Figure 7).

**Figure 8:**
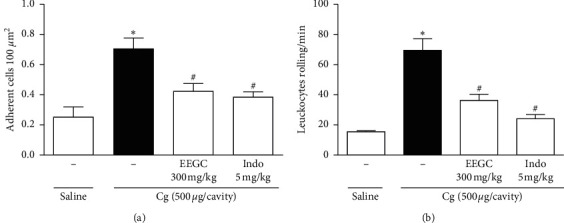
Effect of EEGC on leukocyte rolling (a) and adhesion (b) induced by carrageenan. Mice were orally pretreated with EEGC (300 mg/kg), indomethacin (indo 5 mg/kg), or vehicle. After 60 min, saline or carrageenan was injected i.p. leukocyte rolling, and adhesion was evaluated by intravital microscopy in the mesentery 2 h later. The bars express the mean ± SEM compared with the control vs. treated group; ^#^ or ^*∗*^*P* < 0.001. ^#^Control versus naive; ^*∗*^EEGC versus control. One-way analysis of variance followed by the Newman–Keuls test.

A significant reduction in cell adhesion to the endothelium and in cells rolling provoked by EEGC administration (300 mg/kg) was observed with 40 ± 7% and 48 ± 6% of inhibition, respectively. As expected, the reference drug (indomethacin) at a dose of 5 mg/kg decreased the adhesion (45 ± 5% of inhibition) and consequently the rolling of leukocytes (65 ± 4% of inhibition) (Figures 8(a) and 8(b)).

### 3.4. Effects of EEGC on CFA Inflammatory Model

**Figure 9:**
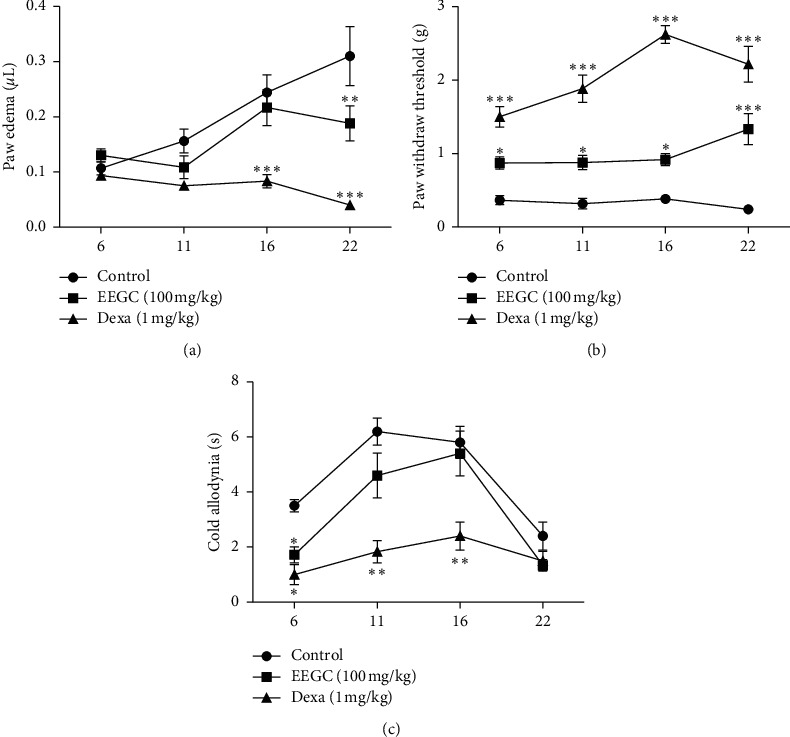
Effect of the oral administration of EEGC on increase in paw edema (a), mechanical sensitivity (paw withdrawal threshold) (b), and cold hypersensitivity (c) in persistent inflammation induced by the CFA model. Mice were treated once/day for 21 days with saline (0.9%, p.o., control), EEGC (100 mg/kg, p.o.), and dexamethasone (1 mg/kg, i.p.) after CFA. The points are expressed as the mean ± SEM compared with the control vs. treated group. ^*∗*^*P* < 0.05; ^*∗∗*^*P* < 0.01 or ^*∗∗∗*^*P* < 0.001. One-way analysis of variance followed by the Newman–Keuls test.

The dose of 100 mg/kg of EEGC was tested in the CFA model of chronic inflammation for 22 days, and the oral EECG treatment was able to reduce significantly the edema volume (a maximal inhibition of 25 ± 18%) after this period. The dexamethasone reference drug showed a reduction in the paw edema on the sixteenth day, when compared to the control group (Figure 9(a)).

EEGC (100 mg/kg) and dexamethasone groups blocked the development of the mechanical hyperalgesia by induced CFA on day 22 of the treatment (Figure 8(b)), while the cold allodynia had an inhibition of sensitivity until the 16^th^ day, possessing its maximum effect both by EEGC (44 ± 21%) and dexamethasone (67 ± 11%) on the sixth day of treatment (Figure 9(c)).

## 4. Discussion

Despite the therapeutic benefit, nonsteroidal anti-inflammatory (NSAIDs) and disease-modifying antirheumatoid drugs have important adverse effects [23], which reinforce the need to search for other safe and efficient therapeutic alternatives. The results of this study contribute with this search showing that EEGC has great antiarthritic and antihyperalgesic potential, corroborating the popular use already reported.

In the carrageenan-induced acute paw edema model, all doses of EEGC exhibited a similar result to the dexamethasone (a reference drug), demonstrating an antiedematogenic potential of this extract. This model is associated with an acute inflammatory process and has several mediators for inflammatory response induction. In the first and second hours, the inflammatory effect is mediated by histamine, serotonin, and kinins, while in the next phase (3 to 6 hours), it is mediated by an increase in the prostaglandin production and COX-2 activation [24, 25]. The EEGC showed great potential in reducing the peripheral inflammatory process, the mechanical hyperalgesia, and the cold allodynia in the paw edema model, actions which may be related to the direct action of the cytokine expression and release of NO in the tissues.

Studies show that the release of proinflammatory cytokines activates the expression of cyclooxygenases, such as COX-1 and COX-2 [26]. These enzymes play an important role in the production of prostaglandins and leukotrienes from arachidonic acid [27]. Several physiological functions such as gastric mucosa protection, regulation of gastric juice release, vascular tone control, and metabolism are related to the action of these molecules [28]. However, physiological effects of COX action such as hyperalgesia, increased body temperature (fever), and inflammatory processes are also found. Studies also show that COX-1 is traditionally known as the constitutive or inducible isoform, while COX-2 is known as inducible isoform in the inflammatory process. The selective COX-2 NSAIDs drugs did not frequently show gastric ulcer induction, which is a common adverse effect observed by traditional NSAIDs. The carrageenan-induced edema was inhibited by EEGC in this study and may be related with an inhibition of the prostaglandin production [29, 30].

The main anti-inflammatory and analgesic drugs used by the population are within the class of NSAIDs. However, the majority of these drugs are not characterized by the selectivity to cyclooxygenases, except for coxibes, which acts in the selective inhibition of COX- 2 [31]. Some studies show that coxibes drugs, after prolonged use, have adverse effects such as direct action on the cardiovascular system [32]. The anti-inflammatory effect was also evaluated in an acute model of pleurisy induced by carrageenan. The carrageenan administration induces the formation of exudate, changes in coloidosmotic pressure, and infiltration of polymorphonuclear leukocytes in the pleural cavity, in addition to the release of proinflammatory mediators [33]. Doses of 700 and 1000 mg/kg of EEGC reduced the total leukocyte migration in the pleural cavity and however did not reduce the protein extravasation.

The antiarthritic activity of EEGC was evaluated by zymosan-induced arthritis in mice. Zymosan is an isolate from the cell wall of the yeast *Saccharomyces cerevisiae* characterized as a polysaccharide that acts in macrophage toll-like 2 (TLR2) receptors and subsequently in the activation of proinflammatory mechanisms [21, 34]. The EEGC decreased the total leukocytes of the articular lavage, indicating a reduction in diapedesis [35].

Our research group identified caffeic acid, ferulic acid, vanillic acid, and catechin in EEGC [8]. Among the biologically active compounds contained in this extract, we point out important anti-inflammatory agents such as caffeic acid, ferulic acid, vanillic acid, and catechin [36] that can be related to the therapeutic effects exhibited by EEGC. Calixto-Campos et al. [37] showed that the anti-inflammatory effect of vanillic acid is related to the inhibition of the neutrophil recruitment and also to the NFkB activation. Vanillic acid can also inhibit the COX-2 and NO expression induced by LPS *in vitro* [38].

Zymosan-induced peritonitis was also evaluated in this study. The adhesion, rolling, and leukocyte migration to the peritoneal cavity were decreased by EEGC, with a similar reduction produced by the reference drug indomethacin. Indomethacin decreases the expression of adhesion molecules such as L-selectin, E-selectin, I-CAM, and VCAM [20, 39]. These molecules play important role in the leukocyte adhesion and in the rolling to the focus of the inflammatory process although others factors are important to the leukocyte migration phenomenon. NO is an important mediator in the leukocyte migration, promoting vasodilation and reducing the recruitment, adhesion, rolling, and leukocyte migration during inflammatory response favoring diapedesis [40, 41]. Since EEGC did not increase the NO levels induced by zymosan, it led us to conclude that the EEGC mechanism of action was not involved in the NO pathway. EEGC maybe act by the same pathway of indomethacin.

Based on the results obtained in acute models, the oral dose of 100 mg/kg EEGC was tested in the CFA model to evaluate EEGC antiarthritic and antihyperalgesic properties. EEGC was effective against mechanical and cold hyperalgesia induced by CFA confirming the popular use of *G*. *celosioides* as an analgesic. In addition, it is possible to report that the mechanical hyperalgesia and cold hyperalgesia processes are characterized as pain indicators since they result from the sensitization and the pain pathway and type C nerve fiber caused by the CFA inflammatory persistent process [42, 43].

In conclusion, the ethanolic extract of *G*. *celosioides* aerial parts showed antiarthritic and antihyperalgesic activities in different evaluated models, decreasing leukocyte recruitment, rolling, adhesion, and migration to the inflammatory focus. Although these results corroborate the popular statement, other studies should be conducted to evaluate the mechanisms of action and to identify the compound responsible for these effects.

## Data Availability

The data used to support the findings of this study are available from the corresponding author upon request.

## Abbreviations

ANOVA: Analysis of variance
C57BL/6: C57 black 6
CFA: Freund's complete adjuvant
COX: Cyclooxygenase
DEXA: Dexamethasone
EDTA: Ethylenediaminetetraacetic acid
EDTA: Ethylenediaminetetraacetic acid
EECG: Ethanolic extract of *Gomphrena celosioides*
ESI-MS: Electrospray ionisation mass spectrometry
ICAM: Intercellular adhesion molecule
IL-1*β*: Interleukin 1*β*
Indo: Indomethacin
NFkB: Factor nuclear kappa B
NO: Nitric oxide
NSAIDs: Nonsteroidal anti-inflammatory
PBS: Phosphate-buffered saline
TLR2: Toll-like 2
TNF: Tumor necrosis factor
VCAM: Vascular cell adhesion molecule.
